# The Effect of Diabetes Mellitus on the Recurrence of Atrial Fibrillation after Ablation

**DOI:** 10.3390/jcm10214863

**Published:** 2021-10-22

**Authors:** Denise Guckel, Khuraman Isgandarova, Leonard Bergau, Misagh Piran, Mustapha El Hamriti, Guram Imnadze, Martin Braun, Moneeb Khalaph, Thomas Fink, Vanessa Sciacca, Georg Nölker, Young-Hee Lee-Barkey, Diethelm Tschöpe, Philipp Sommer, Christian Sohns

**Affiliations:** 1 Clinic for Electrophysiology, Herz- und Diabeteszentrum NRW, Ruhr-Universität Bochum, 32545 Bad Oeynhausen, Germany; dguckel@hdz-nrw.de (D.G.); kisgandarova@hdz-nrw.de (K.I.); lbergau@hdz-nrw.de (L.B.); melhamriti@hdz-nrw.de (M.E.H.); gimnadze@hdz-nrw.de (G.I.); mbraun@hdz-nrw.de (M.B.); mkhalaph@hdz-nrw.de (M.K.); tfink@hdz-nrw.de (T.F.); vsciacca@hdz-nrw.de (V.S.); g.noelker@hospitalverbund.de (G.N.); psommer@hdz-nrw.de (P.S.); 2 Institute for Radiology, Nuclear Medicine and Molecular Imaging, Ruhr-Universität Bochum, 32545 Bad Oeynhausen, Germany; mpiran@hdz-nrw.de; 3 Clinic for Internal Medicine II/Cardiology, Christliches Klinikum Unna Mitte, 59423 Unna, Germany; 4 Diabetes Center, Herz- und Diabeteszentrum NRW, Ruhr-Universität Bochum, 32545 Bad Oeynhausen, Germany; yhlee-barkey@hdz-nrw.de (Y.-H.L.-B.); dtschoepe@hdz-nrw.de (D.T.)

**Keywords:** diabetes mellitus, atrial fibrillation, ablation, pulmonary vein isolation, cryoballoon, fibrosis

## Abstract

Diabetes mellitus (DM) plays a crucial role in the regulation of atrial fibrillation (AF). This study aimed to evaluate the outcome of pulmonary vein isolation (PVI) using a single-shot device in patients with AF and DM. A total of 531 consecutive patients undergoing initial cryoballoon (CB)-guided PVI were evaluated. Two hundred eighty-one patients (53%) suffered from paroxysmal AF (PAF; mean age 51 ± 23.2 years, 26% female), 250 patients (48%) from persistent AF (PERS; 64 ± 10.0 years old, 30% female) and 80 patients (15%) were diagnosed with coincidental DM (68 ± 19.6 years old, 30% female). Follow-up visits were performed at 3, 6 and 12 months including 7-day Holter ECGs. Primary endpoint was the first documented recurrence of atrial tachyarrhythmia. AF recurrence occurred in 26% (140 patients). PAF patients with DM presented with a significantly higher risk for arrhythmia recurrence (Kaplan Meier analysis; Log rank *p* < 0.001 *). Multivariate analyses found DM to be an independent predictor (IP) for AF recurrence (*p* = 0.009 *, hazard ratio (HR) 4.363, confidence interval (CI) 1.456–13.074). In PERS, DM was associated with a 43% increase in AF recurrence (*p* = 0.320, HR 1.427, CI 0.707–2.879). DM has relevant effects on AF recurrence after PVI-only ablation approaches for AF. Major differences were observed in PAF as DM seems to favor the development of individual arrhythmia substrate, which is usually not yet present in PAF. In PERS, DM effects are less pronounced as individual fibrosis has already developed. Thus, personalized paths addressing individual arrhythmia substrates are needed in this specific cohort of patients.

## 1. Introduction

Diabetes mellitus (DM) and atrial fibrillation (AF) are often coinciding diseases and it is well known that DM is a major risk factor for atherosclerotic cardiovascular diseases as well as AF [1,2,3]. AF in patients with DM is associated with increased symptom burden, lower quality of life as well as increased hospitalization and mortality rates [4]. Beyond that, DM seems to be a predictor for freedom from arrhythmia recurrence following AF ablation [5]. Accumulating evidence demonstrates that there might be a complex individual arrhythmia substrate related to structural, electrical, electromechanical and autonomic remodeling. Hitherto, data on complex interactions between AF and DM in terms of atrial fibrosis or scar tissue are scarce [6]. The recent ESC guideline suggests a pre-interventional assessment of potential arrhythmia substrates in the majority of patients as some of these AF patients may require additional ablation beyond PVI [7]. In this context, cardiac magnetic resonance imaging with late gadolinium enhancement (LGE CMR) can help in identifying and subsequently categorizing structural changes inside the left atrial wall [8,9]. However, the impact of DM on the development of an individual and patient-specific arrhythmia substrate is still not completely discovered. Therefore, this study evaluates the outcome and predictors for the success of cryoballoon-guided PVI-only approaches for AF ablation in a cohort of patients with coincidences of AF and DM.

## 2. Methods

This observational study included 531 consecutive patients undergoing index PVI using the 2nd generation 28 mm cryoballoon (Arctic Front Advance, Medtronic, Minneapolis, MN, USA) for catheter ablation of paroxysmal (PAF) and persistent (PERS) AF between January 2013 and February 2020. All patients underwent their index PVI due to recurrent symptomatic episodes of PAF or PERS and failure relative to previous antiarrhythmic drug therapy (AADs).

Patients were divided into two groups (PAF vs. PERS), and the effects of DM on freedom from arrhythmia recurrence were evaluated. The diagnosis of PAF vs. PERS was made using the definition from the ESC guideline for the diagnosis and management of atrial fibrillation [7]. Arrhythmia recurrence was defined as a documented episode of any AF/atrial tachycardia (AT) > 30 s. DM was previously diagnosed by elevated HbA1C levels > 6.5 mg/dL, fasting glucose values > 126 mg/dl and/or pathological oral glucose tolerance testing values accompanied by typical symptoms of DM. Patients at all levels of the DM stage schedule were included. The presence of antidiabetic medication was not an exclusive inclusion criterion.

### 2.1. Periprocedural Management

All patients underwent preprocedural transesophageal echocardiography to rule out thrombus formation inside the LA/LAA. The majority of patients were examined by CMR to evaluate the individual anatomical consideration of the LA and PVs. AADs except for amiodarone were discontinued at least three half-lives before ablation. Anticoagulation with phenprocoumon was continued aiming for an International Normalized Ratio (INR) numbering between 2.0 and 3.0. Direct oral anticoagulants (DOAC) were stopped one half-life before ablation. Pericardial effusion was ruled out immediately after ablation and 4 h later. Anticoagulation was continued within 4 h after the procedure with phenprocoumon or DOAC. AADs were prescribed to the operators’ discretion for a period of 3 months following ablation. Patients stayed in the hospital under continuous rhythm monitoring for at least 36 h.

### 2.2. Ablation Procedure

The procedure was performed under conscious sedation with propofol and analgesia with fentanyl as required.

A quadripolar catheter (Dynamic XT^TM^ Boston Scientific, Marlborough, MA, USA) was used to confirm continuity of the phrenic nerve by pacing in the superior vena cava and continuous abdominal palpation during ablation of the right sided PVs (RPVs). Transseptal puncture was performed guided by intracardiac echocardiography. The cryoballoon was advanced to the LA via a steerable transseptal sheath (Flexcath ^®^Medtronic, Minneapolis, MN, USA). The 28 mm cryoballoon was used in all patients. A multipolar mapping catheter (Achieve^TM^ Mapping Catheter, Medtronic, Minneapolis, MN, USA) was introduced for mapping the PV potentials. The degree of PV occlusion was evaluated by contrast injection after balloon inflation and placement and verified by PV angiography in the initial freezing period. Ablation was performed adherent to a 2*240 s freeze per vein protocol. Adhering to our center specific cryoballoon ablation protocol, the left superior pulmonary vein (LSPV) was isolated initially, followed by the left inferior pulmonary vein (LIPV), the right superior pulmonary vein (RSPV) and the right inferior pulmonary vein (RIPV), respectively. Persistent PVI (entrance and exit block) was confirmed after a waiting period of 20 min.

### 2.3. Follow-Up

After discharge, follow-up visits were scheduled at 3, 6 and 12 months including routine 7-day Holter ECGs and interviews. Unscheduled visits were conducted if required.

### 2.4. Endpoint

We aimed to analyze the impact of DM on freedom from AF/AT recurrence after PVI-only approaches for AF. AF/AT recurrence was judged on ECG documentation and symptoms suggestive for arrhythmia recurrence. Furthermore, we intended to ascertain independent predictors (IPs) of AF/AT-recurrence in this patient cohort allowing for conclusions in terms of personalized paths in AF management in patients with DM.

### 2.5. Data Collection

Data on patients’ characteristics, medication, symptoms and complications were compiled from patients’ records and discharge letters. Procedural parameters and clinical aspects concerning cryoballoon ablation were taken from ablation protocols and procedure related documents. Data were collected retrospectively.

### 2.6. Statistical Analysis

All statistical analyses were performed with SPSS, version 24 (SPSS, Inc., Chicago, IL, USA). All variables were tested for normal distribution. Continuous variables between the groups (PAF and PERS with and without DM) were compared by employing an unpaired two-sided Student’s *t*-test or Mann–Whitney test. Differences in continuous parameters between baseline and follow-up were analyzed by paired Student’s *t*-test or Wilcoxon signed-rank test. Categorical and ordinal data were examined by chi-square, Mann–Whitney tests or Fisher’s exact tests, respectively. Event-free survival was calculated by Kaplan–Meier analysis as time from initial PVI to first documented AF/AT episode > 30 s at the 3, 6 and 12 months follow-up. The log-rank test was used to assess differences in event-free survival time between groups. A Cox proportional hazard regression model was applied to identify IPs of arrhythmia recurrence. Demographic and clinical data from baseline analyses were included in univariate Cox proportional hazard regression models for the primary endpoint. Variables with an unadjusted association with AF/AT recurrence (*p* < 0.1) were analyzed by multivariate Cox regression analysis. Data are presented as mean ± SD or percentage value unless stated otherwise. A *p*-value ≤ 0.05 was considered statistically significant.

## 3. Results

### 3.1. Patients’ Characteristics

The study population consisted of 531 consecutive patients (59 ± 21.2 years old, 28% female) undergoing cryoballoon based ablation for symptomatic AF. Two hundred eighty-one patients (53%) suffered from PAF (51 ± 23.2 years old, 26% female) and 250 patients (47%) from PERS (64 ± 10.0 years old, 30% female). Eighty patients (15%) were diagnosed with DM (68 ± 19.6 years old, 30% female). Depending on the diagnosis of DM, the patients were further divided into PAF patients with DM (23 patients, 9%) and without DM (258 patients, 92%) as well as PERS patients with DM (57 patients, 23%) and without DM (193 patients, 77%).

### 3.2. Baseline Characteristics in PAF and PERS

Baseline characteristics are summarized in Table 1. Severe group specific differences were observed between patients with PAF and PERS (see Table 1). Preprocedural imaging found normal LA and PV anatomy in the majority of patients. In 25 patients (5%), a left common trunk (LCT) was identified, and in 10 cases (<1%) an additional right mid pulmonary vein was (RMPV) identified.

### 3.3. Impact of DM on PAF and PERS

In patients with PAF, several differences were observed between those with and without coincidence of DM (see Table 2). Even more comorbidities were observed in PERS patients with DM in contrast to those without DM (see Table 3).

### 3.4. Procedural Data

Acute procedural success with complete PVI was achieved in all patients. Mean procedural duration (skin-to-skin) was 180 ± 35 min, and mean fluoroscopy time was 18 ± 9 min. Mean nadir temperatures (°C) in PAF patients were −44.6 ± 6.1 °C for the LSPV, −43.2 ± 7.6 °C for the LIPV, −40.1 ± 6.3 °C for LCT, −44.8 ± 6.3° for the RSPV and −43.2 ± 9.1 °C for the RIPV, respectively. Mean nadir temperatures (°C) in PERS were −45.6 ± 6.3 °C for the LSPV, −40.7 ± 5.5 °C for the LIPV, −42.5 ± 13.6 °C for LCT, −47.6 ± 6.8 °C for the RSPV, −45.7 ± 6.4 °C for the RIPV and −38.9 ± 6.1 °C for the RMPV, respectively.

### 3.5. Clinical Outcome

**Figure 1:**
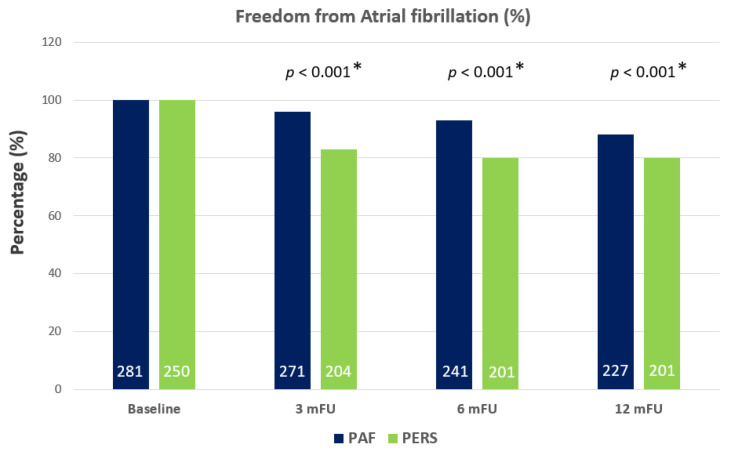
Freedom from AF/AT recurrence (%) in patients with PAF and PERS. AF, atrial fibrillation; AT, atrial tachyarrhythmia; PAF, paroxysmal atrial fibrillation; PERS, persistent atrial fibrillation; mFU, months follow-up; *p*, *p*-value; * indicates statistical significance.

Recurrence of AT/AF occurred in 140 patients (26%) within the follow-up period. Patients with PERS presented with significantly higher recurrence rates compared to PAF patients within the observation period of 12 months. In detail, AF/AT recurrence was significantly higher in patients with PERS 3 months after ablation (18%, 46 patients, *p* < 0.001 *), 6 months after ablation (49 patients, 20%, *p* < 0.001 *) and 12 months (49 patients, 20%, *p* < 0.001 *) after ablation as only 10 PAF patients (4%) suffered from recurrence of AF 3 months after ablation, 40 patients (14%) suffered from recurrence 6 months after ablation and 54 patients (19%) suffered from recurrence 12 months after ablation (see Figure 1).

Recurrence of AT/AF occurred in 140 patients (26%) within 12 m. Ten PAF patients (4%) suffered from recurrence of AF at 3 mFU, 40 patients (14%) suffered from recurrence at 6 mFU and 54 patients (19%) suffered from recurrence at 12 mFU. The recurrence rate of AF was significantly higher in PERS at 3 mFU (18%, 46 patients, *p* < 0.001 *), at 6 mFU (49 patients, 20%, *p* < 0.001 *) and at 12 mFU (49 patients, 20%, *p* < 0.001 *).

Fourty-six PAF patients (57%) were scheduled for re-ablation in consequence of AF recurrence within the observation period of 12 months (PAF + DM, seven patients, 50%; PAF-DM, 39 patients, 48%). Reconnection of at least one PV was documented in all PAF patients. Briefly, PAF patients with DM (seven patients, 50%) were mainly scheduled for cryoballoon ablation (four patients, 57%). Three PAF patients with DM (21%) were treated with radiofrequency (RF) guided re-ablation. One of them received a substrate modification approach based on bipolar low voltage areas.

PAF patients without DM undergoing re-ablation were predominantly treated with repeated cryoballoon-guided ablation (36 patients, 92%). Only a minority of PAF patients without DM scheduled for re-ablation underwent RF-guided catheter ablation (three patients, 8%). None of these patients received additional substrate modification beyond repeat PVI.

Eighteen PERS patients (31%) were scheduled for re-ablation due to AF at the 6 months follow-up or subsequently to their 12 months follow-up (PERS + DM, six patients, 38%; PERS-DM, 12 patients, 28%). Reconnection of at least one PV was documented in 89% (16 patients). All PERS patients suffering from DM (six patients, 38%) underwent RF-guided re-ablation including repeat isolation of the PVs with additional (four patients, 67%) substrate modification or substrate modification alone (two patients, 33%). Seven PERS patients without DM (16%) scheduled for re-ablation underwent RF-guided ablation with re-isolation of single PVs as well. Out of these, four patients (57%) received additional left atrial substrate modification. The remaining PERS patients without DM underwent re-ablation using the cryoballoon (five patients, 12%).

**Figure 2:**
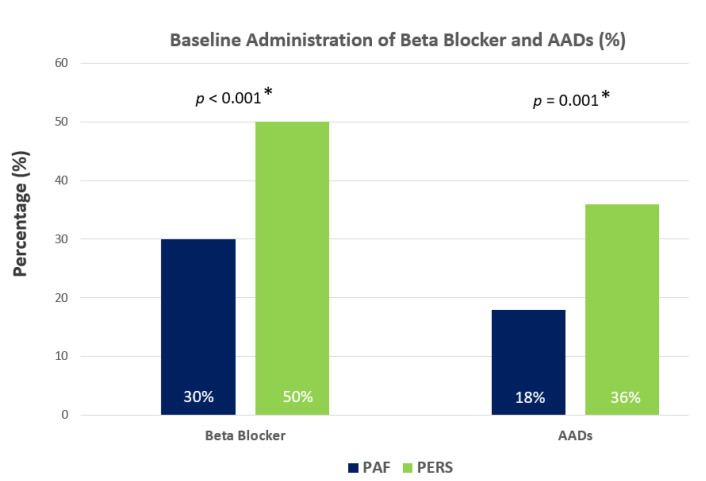
Baseline administration of beta blocker and AADs. AADs, antiarrhythmic agents, PAF, paroxysmal atrial fibrillation, PERS, persistent atrial fibrillation, mFU, months follow-up, *p*, *p*-value, * indicates statistical significance.

Baseline administration of beta blocker was 30% (86 patients) in PAF and 50% (124 patients) in PERS (*p* < 0.001 *). AADs were prescribed in 51 cases (18%) in PAF and in 89 cases (36%) in PERS (*p* = 0.001 *). Thus, far more patients with PERS received these agents compared to PAF patients at baseline (see Figure 2). In the absence of DM, freedom from AF/AT recurrence was significantly higher in patients with PAF (26%, 67 patients) compared to those with PERS (30%, 43 patients) (*p* = 0.024 *). When coincidental DM was present, AF/AT recurrence increased in both subgroups (PAF + DM: 61%, 14 patients; PERS + DM: 28%, 16 patients; *p* = 0.010 *). Eleven percent (*n* = 58) of the patients were lost t follow-up. Baseline characteristics, procedural parameters and proportion of patients with and without DM were not significantly different between those analyzed and those lost to follow-up.

Baseline administration of beta blocker was 30% (86 patients) in PAF and 50% (124 patients) in PERS (*p* < 0.001 *). AADs were prescribed in 51 cases (18%) in PAF and in 89 cases (36%) in PERS (*p* = 0.001 *).

### 3.6. Clinical Outcome in PAF Depending on DM

In patients with PAF, the additional diagnosis of DM was associated with a significantly higher AF/AT recurrence rate (PAF + DM: 61%, *n* = 14 vs. PAF-DM: 26%, *n* = 67; *p* < 0.001 *). At 3 months follow-up, five patients (22%) with PAF and DM presented with AF/AT recurrence compared to five patients (2%) without DM (*p* < 0.001 *). At 6 months follow-up, even 48% (*n* = 11) of PAF patients with DM developed AF recurrence in contrast to 11% (*n* = 29) PAF patients without DM (*p* < 0.001 *). At 12 months follow-up, 52% (*n* = 12) PAF patients with DM suffered from AF recurrence compared to only 16% (*n* = 42) PAF patients without DM (*p* < 0.001 *).

### 3.7. Clinical Outcome in PERS Depending on DM

The additional effect of coincidental DM was lower in PERS compared to PAF. Twenty-seven patients (17%) with PERS have had AF/AT recurrence at 3 months versus 9 patients (24%) of which had additional DM (*p* = 0.097). This was also the case at 6 and 12 months (6 months, PERS-DM: 19% vs. PERS + DM: 22%; *p* = 0.109; 12 months: PERS-DM: 18% vs. PERS + DM: 27%; *p* = 0.175).

### 3.8. Impact of DM and Other Parameters on AF Recurrence

**Figure 3:**
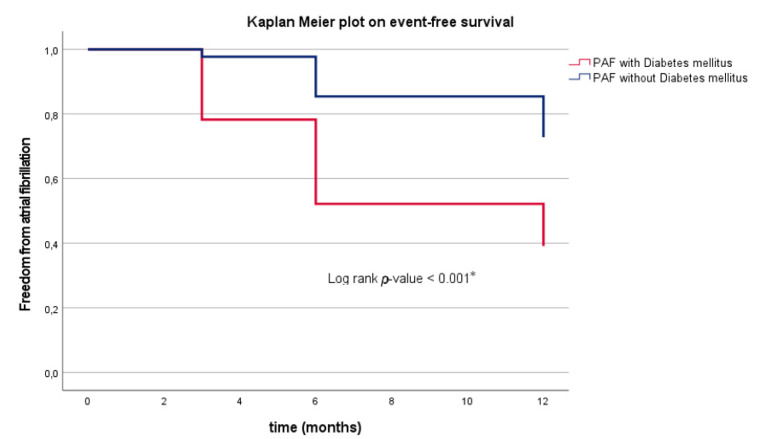
(Representative figure) Kaplan–Meier plot on freedom from AF recurrence in patients undergoing primary PVI due to PAF. AF, atrial fibrillation; PVI, pulmonary vein isolation; PAF, paroxysmal atrial fibrillation; DM, diabetes mellitus; FU, follow-up. A *p*-value ≤ 0.05, * and bold letters indicate statistical significance.

**Figure 4:**
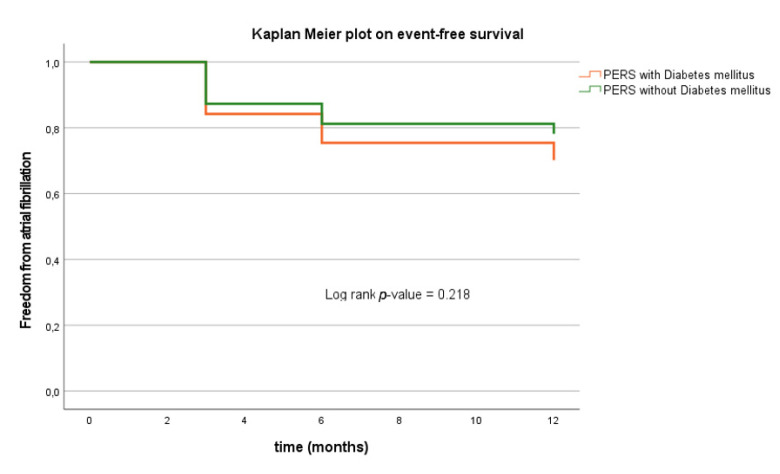
Kaplan–Meier plot on freedom from AF recurrence in patients undergoing primary PVI ablation due to PERS. AF, atrial fibrillation; PVI, pulmonary vein isolation; PERS, persistent atrial fibrillation; DM, diabetes mellitus; FU, follow-up.

The estimated risk for AF/AT recurrence was significantly higher when patients with PAF have had additional history of DM (log-rank *p* < 0.001 *; Table 2; Figure 3). Multivariate Cox regression analysis identified DM (*p* = 0.010 *, hazard ration (HR) 4.363, confidence interval (CI) 1.456–13.074) as IP for AF recurrence in the PAF cohort. Supplementary Table S1 demonstrates that there is a >4-fold higher risk for AF/AT recurrence due to coincidence of DM. Univariate cox regression analysis found that DM was associated with a 43% higher risk for AF/AT recurrence in PERS patients (*p* = 0.320, HR 1.427, CI 0.707–2.879). In addition, Kaplan Meier analysis verified a lower estimated risk for AF/AT recurrence in PERS without DM compared to PERS with DM without reaching the level of statistical significance (log-rank *p* = 0.218) (Figure 4). Multivariate Cox regression analysis revealed the male gender as strong IP for AF/AT-freedom (*p* = 0.017 *, HR 0.477, CI 0.260–0.875) in patients with PERS (Supplementary Table S2). Referring to the administration of beta blocker and AADs, significant differences were observed depending on the diagnosis of PAF or PERS (Figure 2). No conclusions could be drawn regarding an association between antidiabetic therapy and AT/AF recurrence freedom, neither in PAF (*p* = 0.098) nor in PERS (*p* = 0.070).

### 3.9. Impact of Baseline Parameters on AF Recurrence in DM Patients

On the basis of Cox regression analyses, there was no statistical evidence that individual baseline parameters of DM patients were predictive for recurrence of AF (Supplement Table S3).

Patients with DM showed a significantly higher recurrence rate of AF in the FU in comparison to patients without DM (log-rank *p*-value < 0.001 *).

## 4. Complications

Major complications, requiring intervention, occurred in seven patients (1%) irrespective of the diagnosis of DM. These complications consisted of two patients with phrenic nerve injury, two patients with pericardial tamponade, one patient with stroke and two patients with inguinal venous bleedings and the need for transfusion.

## 5. Discussion

### 5.1. Main Findings

This study aimed to evaluate the freedom from any AF/AT recurrence after cryoballoon-guided PVI in patients with DM and to address the issue of whether there are certain individual risk factors for arrhythmia recurrence in this preselected cohort of patients with coincidence of DM and AF.

This study has four major findings: First, DM has relevant effects on AF/AT recurrence after cryoballoon-guided PVI. Second, DM was revealed as IP for AF recurrence in PAF. Third, the coincidence of PAF and DM results in an early stage of atrial cardiomyopathy as a potential substrate for AF with comparable ablation effects as we found in more chronic stages of AF. Fourth, the preprocedural assessment of individual arrhythmia substrates should be performed in all patients with AF and DM, and patients should be scheduled for cryoballoon-guided PVI-only approaches or RF-guided ablation (allowing for additional substrate modification) based on this multimodal evaluation.

### 5.2. Impact of DM on Arrhythmia Recurrence

DM has been described as an important cardiovascular risk factor. Data from the Framingham Heart Study revealed that DM is linked to an increased prevalence of AF [2]. While some smaller studies failed to prove an association between DM and AF [10], a meta-analysis and some case-control studies stated a 34% higher risk for AF in patients with coincidence of DM [6].

Preclinical data have already described the negative synergistic effects of AF and DM in terms of cellular remodeling and fibrosis [11], but there is still the need of evidence for data reporting on the impact of DM on individual arrhythmia substrates and the outcome of AF ablation.

In this observational study, 80 patients (15%) were diagnosed with coincidences of DM and symptomatic AF. Our data highlight a significant effect of DM on freedom from any AF/AT recurrence after PVI using the CB in patients with PAF (log-rank *p*-value < 0.001 *; Figure 3). Moderate adverse effects of DM on arrhythmia recurrence following ablation were also observed in PERS (Figure 4). The complex pathology of DM is associated with cardiac remodeling resulting in structural atrial alterations [6]. In contrast to PAF, PERS patients present with more distinct cardiac atrial remodeling processes and fibrotic changes. As additional effects of DM might not be the key player for the origin and genesis of arrhythmia substrates in PERS, minor effects of DM were probably observed in PERS compared to PAF.

Patients with DM present with a slightly higher recurrence rate of AF in the FU in comparison to patients without DM without reaching statistical significance (log-rank *p*-value = 0.220).

Concerning ablation aspects, the impact of scarred or fibrotic tissue on arrhythmia recurrence has been demonstrated in other study collectives. As supported by the CASTLE AF clinical trial (NCT00643188) and data from the DECAAF study (NCT02529319), the formation of fibrosis seems to be a powerful predictor of arrhythmia free survival after ablation [12,13]. As DM results in cellular and structural changes even at this relative early stage of AF, one may speculate that the effect of a pure PVI approach might probably not be sufficient to target the complete arrhythmia substrate. One may suggest that these patients might benefit from high density mapping or pre-procedural LGE CMR in order to visualize potential individual arrhythmia substrates allowing customized lesion sets and substrate modification [9].

Striving for ideal ablation strategies in which not only pure categorization of the type of AF (PAF or PERS) but also individual risk factors, such as DM, should be considered.

Beyond that, an early intervention might prevent increased stages of atrial cardiomyopathy and remodeling [14]. This becomes even more important since the EAST-AFNET 4 trial (NCT01288352) suggested early rhythm control to prevent AF patients with concomitant risk factors from cardiovascular death and stroke [15].

**Figure 5:**
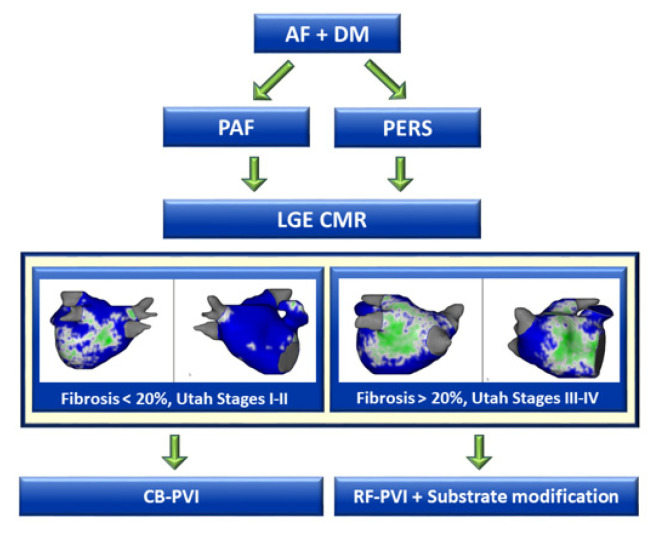
Proposed decision pathway for patients with DM scheduled for ablation due to AF. DM, diabetes mellitus, AF, atrial fibrillation, LGE CMR, Cardiac magnetic resonance imaging with late gadolinium enhancement; PAF, paroxysmal atrial fibrillation; cryoballoon-PVI, cryoballoon pulmonary vein isolation; RF-PVI, radiofrequency pulmonary vein isolation; PERS, persistent atrial fibrillation.

Taking all of these observations into account, our data clearly address the need for personalized paths in arrhythmia management in patients with DM and AF. In our opinion, CMR might be a very useful tool when striving for an individual decision pathway (Figure 5).

All patients with DM and AF scheduled for an ablation procedure undergo LGE CMR in order to identify and categorize structural changes inside the left atrial wall irrespective of the diagnosis of PAF or PERS.

Patients with an amount of fibrosis < 20% (Utah stages I–II) are planned for cryoballoon-PVI, whereas patients with areas of fibrotic tissue accounting for >20% (Utah stages III-IV) receive RF-PVI with additional substrate modification.

### 5.3. Additional Predictors of AF Recurrence

#### 5.3.1. Baseline Characteristics

With regards to patients’ baseline characteristics, significant differences were found based on the diagnosis of DM and the type of arrhythmia (PAF vs. PERS). These individual characteristics might have a direct impact on AF ablation and freedom from AF/AT recurrence (Table 1, Table 2 and Table 3). With the exception of gender aspects and DM, all other baseline parameters could be ruled out as IPs for AF-recurrence (Supplement Tables S1 and S2).

#### 5.3.2. B-Blocker and Antiarrhythmic Agents

By focusing on drug therapy, the administration of beta blocker and AADs was significantly lower in PAF (30% beta blocker and 18% AADs) compared to PERS (50% beta blocker and 36% AADs) (Figure 2). The rate of AADs in PAF is in line with previous data from AF cohorts [16,17]. In contrast, the administration of AADs was found to be higher (*p* = 0.001 *) in PERS vs. PAF most likely due to the expectation of more severe additional arrhythmia substrates beyond PV triggers in line with increased arrhythmia recurrence rates in PERS. The intensified use of beta blocker and AADs might also be one reason for the observed rather moderate differences in terms of AF recurrence in PERS vs. PAF irrespective of the coincidence of DM (Figure 1).

#### 5.3.3. Metabolic Parameters

As expected, patients diagnosed with DM presented with significantly altered metabolic parameters (Table 1 and Supplementary Table S1). PERS patients with DM had significantly elevated triglyceride-levels and lower HDL-values reflecting their impaired metabolic status (Table 3). As these adverse metabolic variations might trigger atrial remodeling, more severe effects might be detected in PERS as well as in PAF patients with DM compared to those without. This might also be a possible explanation for the limited success rate of a PVI-only approach using the cryoballoon in the case of PERS or PAF with concomitant DM. Previous studies reported on wide circumferential ablation strategies being associated with a more desirable clinical outcome in PAF and PERS [18,19].

#### 5.3.4. Gender Disparities and the Autonomic Nervous System

Interestingly, the male gender was confirmed as predictive for AF/AT-freedom in PERS with an up to 53% risk reduction for AF recurrence in comparison to the female gender (Supplementary Table S2).

Gender disparities in patients scheduled for cryoballoon-guided AF ablation have also been reported from a previous study in patients with PERS [17]. In line with our previous data, women seem to be at a higher risk for AF/AT recurrence; this is possibly due to a higher affinity for cardiac remodeling as well as adverse alterations in heart rate variability (HRV) resulting in a predominance of sympathetic tone [17]. Beyond that, PVI associated vagal reactions reflecting cardiac intrinsic ANS modulation were found to be strong IPs for AF-free survival [17], which underlines the important role of the autonomic nervous system (ANS) in the initiation and maintenance of AF. DM seems to have an unfavorable impact on HRV too [20]. Concerning these aspects in detail, further studies are required.

#### 5.3.5. Predictive Value of Baseline Parameters on AF Recurrence in DM Patients

Individual baseline parameters of DM patients were not predictive for AF recurrence (Supplementary Table S3); on the other hand, the combination of different risk factors than one risk factor alone seems to cause complex metabolic changes that result in cardiac remodeling processes and, thus, favor the development of arrhythmia substrates.

### 5.4. Clinical Perspective and Translational Outlook

Due to the impact of DM on cardiovascular morbidity and mortality including effects on initiation and perpetuation of AF and cryoballoon ablation outcomes, further studies focusing on modifications of treatment strategies for DM are required. With respect to remodeling aspects, the influence of specific biomarkers associated with the development of atrial fibrosis has to be analyzed, and these effects need translation into clinics. Moreover, the impact of blood glucose control on the presence of AF as well as arrhythmia recurrence following ablation has to be further evaluated. Preprocedural LGE CMR of the LA allowing for individual ablation approaches should be considered in patients with DM and AF.

## 6. Conclusions

DM has relevant effects on arrhythmia recurrence after cryoballoon-guided PVI. Our data demonstrates a stronger ablation-induced effect in patients with PAF. Major differences were observed in PAF as DM seems to favor the development of individual arrhythmia substrate, which is usually not yet present in PAF. In PERS, DM effects are less pronounced as individual fibrosis has already developed. Thus, personalized paths addressing individual arrhythmia substrates are needed in this specific cohort of patients.

## Figures and Tables

**Table 1:** Baseline Characteristics.

Characteristics	PAF (*n* = 258)	PERS (*n* = 193)	*p*-Value
Age (years)	51.0 ± 23.2	65.0 ± 10.0	<0.01 *
Gender, female	66 (26%)	57 (30%)	0.02 *
BMI (kg/m^2^)	26.8 ± 3.8	29.0 ± 5.3	<0.01 *
LVEF (%)	54.1 ± 4.4	54.3 ± 2.8	0.06
LA diameter (mm)	38.2 ± 6.5	43.6 ± 8.5	<0.01 *
CMP	22 (9%)	21 (11%)	0.06
CAD	14 (4%)	11 (6%)	0.09
Hypertensive CMP	8 (3%)	7 (4%)	0.11
H(O)CM	0 (0%)	2 (1%)	0.40
Valvular CMP	0 (0%)	1 (1%)	0.64
Hypertension	98 (38%)	122 (63%)	<0.01 *
Smoking	26 (10%)	38 (20%)	<0.01 *
Beta blocker BL	86 (30%)	124 (50%)	<0.01 *
AADs BL	51 (18%)	89 (36%)	<0.01 *

Continuous variables are shown as the mean ±SD and categorical variables as the number (%). A *p*-value ≤ 0.05, * and bold letters indicate statistical significance. PAF, paroxysmal atrial fibrillation; PERS, persistent atrial fibrillation; BMI, body mass index; LVEF, left ventricular ejection fraction; LA, left atrium; CMP, cardiomyopathy; CAD, coronary artery disease; H(O)CM, hypertrophic (obstructive) cardiomyopathy; BL, baseline; AADs, antiarrhythmic agents.

**Table 2:** Baseline Characteristics in PAF depending on DM.

Characteristics	DM (*n* = 23)	No DM (*n* = 258)	*p*-Value
Age (years)	58.1 ± 28.3	50.8 ± 22.9	0.03 *
Gender, female	7 (30%)	66 (26%)	0.01 *
BMI (kg/m^2^)	30.0 ± 5.1	27.0 ± 3.8	<0.01 *
LVEF (%)	52.1 ± 5.7	54.1 ± 4.4	0.13
LA diameter (mm)	40.2 ± 7.7	38.2 ± 6.5	0.07
CMP	2 (9%)	22 (9%)	0.25
CAD	2 (9%)	14 (4%)	0.26
Hypertensive CMP	0 (0%)	8 (3%)	0.44
H(O)CM	0 (0%)	0 (0%)	1.00
Valvular CMP	0 (0%)	0 (0%)	1.00
Hypertension	19 (83%)	98 (38%)	<0.01 *
Smoking	8 (35%)	26 (10%)	<0.01 *
Beta blocker BL	4 (17%)	82 (32%)	<0.01 *
AADs BL	5 (22%)	46 (18%)	0.02 *
HbA1c (%)	6.2 ± 0.4	5.7 ± 0.7	0.01 *
Cholesterol (mg/dL)	179.3 ± 36.0	207.4 ± 40.9	0.06
Triglyceride (mg/dL)	187.25 ± 77.8	148.7 ± 94.8	0.21
LDL (mg/dL)	114.5 ± 18.5	127.6 ± 34.1	0.15
HDL (mg/dL)	60.0 ± 37.8	53.4 ± 28.7	0.64
CRP (mg/dL)	0.6 ± 0.8	0.5 ± 0.9	0.59

Continuous variables are shown as the mean ± SD and categorical variables as the number (%). A *p*-value ≤ 0.05, * and bold letters indicate statistical significance. PAF, paroxysmal atrial fibrillation; DM, diabetes mellitus; BMI, body mass index; LVEF, left ventricular ejection fraction; LA, left atrium; CMP, cardiomyopathy; CAD, coronary artery disease; H(O)CM, hypertrophic (obstructive) cardiomyopathy; BL, baseline; AADs, antiarrhythmic agents; LDL, low-density lipoprotein; HDL, high-density-lipoprotein; CRP, C-reactive-protein.

**Table 3:** Baseline Characteristics in PERS depending on DM.

Characteristics	DM (*n* = 57)	No DM (*n* = 193)	*p*-Value
Age (years)	70.4 ± 9.9	65.3 ± 9.8	<0.01 *
Gender, female	18 (32%)	57 (30%)	0.09
BMI (kg/m^2^)	30.7 ± 4.7	28.9 ± 5.3	0.03 *
LVEF (%)	53.2 ± 3.6	54.3 ± 2.8	0.04 *
LA diameter (mm)	44.3 ± 4.5	43.7 ± 8.5	0.43
CMP	15 (26%)	4 (11%)	<0.01 *
CAD	9 (16%)	11 (6%)	0.03 *
Hypertensive CMP	6 (11%)	7 (4%)	0.05 *
H(O)CM	0 (0%)	2 (1%)	0.66
Valvular CMP	0 (0%)	1 (1%)	0.81
Hypertension	49 (86%)	122 (63%)	<0.01 *
Smoking	17 (30%)	38 (20%)	0.05 *
Beta blocker BL	46 (82%)	146 (76%)	0.07
AADs BL	23 (40%)	66 (34%)	0.04 *
HbA1c (mg/dL)	6.7 ± 1.2	5.5 ± 0.3	<0.01 *
Cholesterol (mg/dL)	179.4 ± 44.8	208.2 ± 44.5	0.05 *
Triglyceride (mg/dL)	181.08 ± 91.8	150.9 ± 85.9	<0.01 *
LDL (mg/dL)	105.6 ± 36.4	130.1 ± 35.9	<0.01 *
HDL (mg/dL)	47.6 ± 21.1	52.2 ± 16.4	<0.01 *
CRP (mg/dL)	0.6 ± 0.8	0.5 ± 0.9	0.59

Continuous variables are shown as the mean ± SD and categorical variables as the number (%). A *p*-value ≤ 0.05, * and bold letters indicate statistical significance. PERS, persistent atrial fibrillation; DM, diabetes mellitus; BMI, body mass index; LVEF, left ventricular ejection fraction; LA, left atrium; CMP, cardiomyopathy; CAD, coronary artery disease; H(O)CM, hypertrophic (obstructive) cardiomyopathy; BL, baseline; AADs, antiarrhythmic agents LDL, low-density lipoprotein; HDL, high-density-lipoprotein; CRP, C-reactive-protein.

## Data Availability

The data underlying this article will be shared upon reasonable request to the corresponding author.

## Supplementary Materials

The following are available online at https://www.mdpi.com/article/10.3390/jcm10214863/s1, Table S1: Cox regression analyses of risk factors for recurrence of AF in PAF patients undergoing 2G-CBA. Table S2: Cox regression analyses of risk factors for recurrence of AF in PERS patients undergoing 2G-CBA. Table S3: Cox regression analyses of risk factors for recurrence of AF in DM patients undergoing 2G-CBA.
