# Supplementary material for: The Effect of Diabetes Mellitus on the Recurrence of Atrial Fibrillation after Ablation
Source: J Clin Med. 2021 Oct 22;10(21):4863. doi: 10.3390/jcm10214863 (PMC8584917; doi:10.3390/jcm10214863)
Supplement: Supplementary file 1 [file jcm-10-04863-s001.zip › jcm-1404467-supplementary.pdf]

Supplementary Table S1. Cox regression analyses of risk factors for recurrence of AF in PAF patients undergoing 2G-CBA.

Risk Factor	Hazard Ratio	95% Lower	95% Upper	*p*-Value
Age (years)	0.989	0.942	1.038	0.64
Gender, male	2.696	0.351	20.723	0.34
BMI (kg/m^2)	1.019	0.916	1.132	0.73
LVEF (EF%)	0.978	0.936	1.022	0.33
LA diameter (mm)	1.042	0.984	1.104	0.16
Hypertension	0.761	0.146	3.964	0.75
Cardiomyopathy	0.680	0.111	4.174	0.68
Diabetes mellitus	4.363	1.456	13.074	**0.01** *
AADs Baseline	1.212	0.947	1.346	0.24
Smoking	1.460	0.314	6.783	0.63

A *p*-value ≤ 0.05 * and bold letters indicate statistical significance. BMI, body mass index; LVEF, left ventricular ejection fraction; LA, left atrium; AADs, antiarrhythmic agents.

Supplementary Table S2. Cox regression analyses of risk factors for recurrence of AF in PERS patients undergoing 2G-CBA. A *p*-value ≤ 0.05*and bold letters indicate statistical significance. BMI,

Risk Factor	Hazard Ratio	95% Lower	95% Upper	*p*-Value
Age (years)	0.997	0.965	1.031	0.87
Gender, male	0.477	0.260	0.875	**0.02** *
BMI (kg/m^2)	0.980	0.926	1.037	0.48
LVEF (EF%)	0.972	0.887	1.066	0.55
LA diameter (mm)	1.022	0.977	1.070	0.34
Hypertension	0.644	0.338	1.228	0.18
Cardiomyopathy	0.573	0.223	1.472	0.25
Diabetes mellitus	1.427	0.707	2.879	0.32
AADs Baseline	1.417	0.901	2.228	0.13
Smoking	1.574	0.931	2.247	0.12

body mass index; LVEF, left ventricular ejection fraction; LA, left atrium; AADs, antiarrhythmic agents.

Supplementary Table S3. Cox regression analyses of risk factors for recurrence of AF in DM patients undergoing 2G-CBA.

Risk Factor	Hazard Ratio	95% Lower	95% Upper	*p*-Value
Age (years)	1.015	0.973	1.060	0.48
Gender, male	0.597	0.256	1.391	0.23
BMI (kg/m^2)	1.021	0.916	1.138	0.71
LVEF (EF%)	0.971	0.861	1.096	0.63
LA diameter (mm)	1.009	0.920	1.108	0.84
Hypertension	0.399	0.145	1.093	0.07
CMP	1.448	0.610	3.433	0.40
Smoking	1.284	0.504	3.273	0.60
HbA1c (mg/dl)	1.008	0.64	1.404	0.24
LDL (mg/dl)	0.998	0.981	1.016	0.87

A *p*-value ≤ 0.05 * and bold letters indicate statistical significance. AF, atrial fibrillation; DM, diabetes mellitus; BMI, body mass index; LVEF, left ventricular ejection fraction; LA, left atrium; CMP, cardiomyopathy; LDL, low-density lipoprotein.
